# Radiosurgery to the Postoperative Tumor Bed for Metastatic Carcinoma Versus Whole Brain Radiation After Surgery

**DOI:** 10.7759/cureus.885

**Published:** 2016-11-19

**Authors:** Kristen Scheitler-Ring, Bin Ge, Greg Petroski, Gregory Biedermann, N. Scott Litofsky

**Affiliations:** 1 Division of Neurological Surgery, University of Missouri School of Medicine; 2 Office of Medical Research, University of Missouri School of Medicine; 3 Division of Radiation Oncology, University of Missouri School of Medicine

**Keywords:** stereotactic radiosurgery, whole brain radiation therapy, metastatic carcinoma, surgery, tumor bed

## Abstract

**Background:**

The treatment paradigm from postoperative whole brain radiation therapy (WBRT) to post-operative stereotactic radiosurgery (SRS) to the tumor bed has shifted with little data to evaluate whether each treatment modality confers equivalent tumor control and survival outcomes.

**Methods:**

Patients with surgical resection of single brain metastases from January 2010 to December 2014 were treated postoperatively with either WBRT or SRS. Retrospective patient data was compared for local control, distant brain recurrence, overall survival, and radiation complications.

**Results:**

Forty-six received WBRT, and 37 received tumor bed SRS. Twelve of 35 (34%) SRS patients experienced local recurrence compared to 17 of 31 (55%) WBRT patients (p = 0.09). The median survival was 440 days (14.7 months) for SRS and 202 days (6.7 months) for WBRT (p = 0.062, log-rank). SRS demonstrated improved survival benefit in the first six months (p = 0.0034; Wilcoxon). Radiation-related adverse changes after SRS (22%) were not statistically different from WBRT (8.7%) (p = 0.152). Age (p = 0.08), systemic cancer status (p = 0.30), Graded Prognostic Assessment (p = 0.28), number of brain metastases at diagnosis (p = 0.65), tumor volume at diagnosis (p = 0.13), new brain lesions (p = 0.74) and neurologic versus systemic cause of death (p = 0.11) did not differ between the groups.

**Conclusions:**

Following surgical resection, tumor bed SRS can be used effectively in lieu of WBRT to treat brain metastases with comparable local control and distant control and without significantly more adverse events.

## Introduction

Traditionally, brain metastases have been treated with surgical resection followed by whole brain radiation therapy (WBRT) to decrease the rates of local recurrence, distant brain recurrence and neurologic cause of death [1-2]. Since it treats the entire brain, WBRT is thought to control tumor progression by treating identifiable metastases and otherwise unidentifiable micrometastases. However, WBRT has become increasingly implicated with global cognitive impairment that persists after cessation of treatment [3-4], thereby expanding concerns regarding quality of life despite reducing tumor burden. Furthermore, WBRT may not increase overall survival time [5-6]. Consequently, shifts in the treatment paradigm for brain metastases from postoperative WBRT to postoperative stereotactic radiosurgery (SRS) to the tumor bed are occurring [7].

SRS delivers high-dose radiations to a discrete volume within the brain. Because SRS spares healthy brain parenchyma, it confers a theoretically favorable alternative to WBRT and is being increasingly utilized in the management of brain metastases. SRS confers less cognitive decline in patients relative to WBRT [5, 8-10] and improved quality of life [11]. A number of studies have examined the ability of SRS to the post-resection tumor bed to control metastatic brain disease after tumor resection [12-27]. All of these studies, however, look only at populations of patients treated with SRS and do not compare outcomes between postoperative WBRT and SRS. Questions remain as to whether tumor bed SRS imparts long-term survival advantages equivalent to those of WBRT [9].

Based on these gaps in the understanding of the potential benefits of SRS to the post-resection tumor bed, we sought to compare the outcomes of patients who received surgical resection followed by either WBRT or SRS for brain metastases. We hypothesized that the outcomes related to tumor control of tumor-bed SRS would be clinically equivalent to those of postoperative WBRT without an increase in complications.

## Materials and methods

### Patient data

All patients who received either surgical resection followed by WBRT or SRS for a presumed metastatic brain tumor from January 1, 2010 to December 31, 2014 at the University Hospital were retrospectively identified from patient treatment databases of the senior author [National Science Library (NSL)]. Patients were excluded if they were from the Veterans' Administration system or state prisoners (due to different Institutional Review Board processes), if they had no documentable follow-up after radiation treatment, or if they died within three months of treatment.

Waiver of consent was granted by the University of Missouri Health Sciences Institutional Review Board (IRB #1210881). Electronic medical records were reviewed for variables collected at the time of diagnosis, including age, sex, status of systemic disease outside of the brain (no evidence, stable, or progressive), number of brain metastases, site of primary malignancy (lung, breast, melanoma, colorectal, other), and tumor volume. Graded Prognostic Assessment (GPA) [28] was also determined. The data collected following treatment included chemotherapy received (yes or no), development of adverse radiation changes, management of adverse radiation changes (observation, steroids, or surgery), same site recurrence, distant recurrence within the central nervous system (CNS), and cause of death (neurologic or systemic).

Once the data was collected and collated, SRS patients were compared to WBRT patients for the outcome measures of local control, new brain metastases, overall survival, and adverse radiation changes.

### Radiation treatment

Patients were chosen for SRS or WBRT at the discretion of the treating radiation oncologist and neurosurgeon in a non-randomized fashion. 

All SRS patients were treated with SRS using the Varian Trilogy TX linear accelerator system (Varian, Palo Alto, CA) at the Ellis Fischel Cancer Center, part of the University of Missouri Health System. Each patient underwent neuronavigational magnetic resonance imaging (MRI). In addition, the patient was immobilized with an Aquaplast Mask (Civco, Coralville, IA) which was attached to the couch. When appropriate, the patient was administered intravenous (IV) contrast dye, and axial images were obtained throughout the head for the planning computed tomography (CT) scan. Initially a bite block was constructed as a part of the immobilization apparatus, but this was discontinued in 2013 in favor of using Vision RT (London, UK). The planning CT scan was fused to the planning magnetic resonance image (MRI). The gross tumor volume (GTV) was contoured to the leading edge of the enhancement on gadolinium-enhanced T1-weighted axial MRI images at the site of the previously defined tumor for which the surgery was performed. The treatment plan was then optimized by dosimetry and physics using Varian Eclipse (Varian, Palo Alto, CA). Each plan used seven or eight static fields with a mini-multileaf collimator with 2.5 mm leaves. The prescription tumor volume (PTV) included a 2 mm expansion of the GTV as is usually done with frameless SRS [12-14, 16, 18, 25-27]. The dose to the margin was 1500 centigray (cGy) prescribed to the 80% isodose line for single fraction SRS. If the spherical diameter equivalent of the GTV exceeded 3 cm, then fractionated radiosurgery was performed in which the dose to the margin was 2100 cGy prescribed in three fractions to the 80% isodose line. Dose constraints to critical structures for single fraction SRS included the following: eyes - 800 cGy, optic nerves - 800 cGy, optic chiasm - 800 cGy, and brainstem - 1000 cGy.

The treatment plans were reviewed by the radiation oncologist and the neurosurgeon. Prior to each treatment, quality assurance was performed by physics on the multi-leaves, isocenter and cone beam. Immediately prior to each treatment, a cone beam CT was performed and compared to the reference CT and adjustments if any were made. A six degrees of freedom couch (Civco, Coralville, IA) was added when the Vision RT system was installed, which allowed for rotational adjustments of the couch at the time of treatment. Using the Vision RT system, the patient’s position during treatment delivery was monitored.

For whole brain radiation therapy, treatments were performed at Ellis Fischel Cancer Center or a nearby local community hospital. All patients were immobilized using the Aquaplast Mask. CT images were obtained for simulation. Typically, treatments were delivered using lateral or anterior oblique fields using photons. The doses were 3000 cGy delivered in 10 or 12 fractions.

### Definitions

The status of systemic disease was defined on the basis of computed tomography/positron emission tomography (CT/PET) of the body or CT of the chest/abdomen/pelvis. Cause of death was defined as neurologic if the patient suffered progressive neurological deficits or symptoms indicative of elevated intracranial pressure with stable systemic disease; otherwise, the cause of death was defined as systemic (e.g. the patient died from infection, widespread tumor burden, cardiac events, or unknown). Time to treatment was measured between the date of surgery and the date of the first SRS or WBRT treatment. Same site recurrence was defined as the appearance of new enhancement or tumor growth in or immediately around the previously resected tumor bed on follow-up MRI, and distant recurrence was defined as evidence of tumor recurrence elsewhere within the brain. Adverse radiation events were defined as increased cerebral edema and enhancement after treatment with brain PET/CT indicative of a hypometabolic lesion with improvement on subsequent follow-up without surgical intervention or with pathological confirmation of radiation necrosis.

### Statistical analysis

Summary statistics were used to assess patients’ characteristics. Means and standard deviations or medians were compared for numerical characteristics, such as age and number of metastases. Frequency distributions were compared for categorical characteristics such as sex, status of systemic disease, neurologic cause of death, chemotherapy treatment, and cancer primary. Two-sample t-tests were conducted to compare numerical characteristics between groups. Chi-square or Fisher's Exact tests were used to compare categorical characteristics between groups.

The Wilcoxon rank-sum test was used to test differences in tumor volume between groups. A log-normal survival model was used to assess the relationship between survival time and tumor volume or number of metastases. Wilcoxon rank-sum tests were used to assess the relationship between tumor volume and dichotomous outcomes, such as same site recurrence, distant recurrence, and radiation changes. The Mann-Whitney U-test was performed to assess the relationship between graded prognostic assessment and treatment type selected.

Overall survival (OS) was computed using the Kaplan-Meier method, and survival distributions were compared using a multivariate regression model adjusting for the following patient characteristics: age, sex, and status of systemic disease. A log-normal survival model was ultimately selected for this comparison.

The following binary outcomes were compared using the Chi-square test: same site recurrence, distant recurrence, and radiation changes. Odds ratios and 95% confidence intervals were reported. Due to concern that individuals who died within three months of surgical resection would skew results because of their presumed more severe disease, these patients were excluded from analysis of binary outcomes as previously stated. All 13 patients were treated with WBRT.

Data was analyzed using statistical software SAS 9.4 (SAS Institute Inc. Cary, NC, USA). A p-value of less than 0.05 was considered statistically significant.

## Results

### Patient demographics

Following surgery, 37 patients received SRS to the resection bed and 46 patients received WBRT. Age (p = 0.08), systemic cancer status (p = 0.30), number of brain metastases at diagnosis (p = 0.65), tumor volume at diagnosis (p = 0.13), chemotherapy regimen (p = 0.18), development of new brain lesions (p = 0.74), and neurologic versus systemic cause of death (p = 0.11) did not differ between the groups. GPA was not different between the groups (p=0.28). In both the groups, the most common primary was non-small cell lung cancer (NSCLC), followed by breast cancer and melanoma (Table 1). The tumor type also did not differ between the groups (p = 0.26).

**Table 1:** Baseline characteristics stratified by treatment type

	Surgery + SRS (n = 37)	Surgery + WBRT (n = 46)	p-Value
Age (mean ± SD)	63 ± 12	59 ± 8.5	0.080
Female	46%	57%	0.34
Number of metastases (median)	1.00	1.00 (n=41)^b^	0.65
Tumor volume (cm^3^) (mean + SD)	14.8 ± 8.5	19.5 ± 13.6	0.13
Time to treatment	20.5 ± 18.3	26.5 ± 10.5	
Status of systemic disease			0.34
* No evidence*	11%	24%	
* Stable*	22%	17%	
* Progressive*	68%	59%	
Neurological cause of death	43%	66%	0.11
Grade Prognostic Assessment	2.5 ± 0.67	2.3 ± 0.70	0.28
Chemotherapy received	91%	80%	0.18
Cancer primary			0.26
* NSCLC*	51%	52%	
* SCLC*	0%	7%	
* Breast*	14%	15%	
* Melanoma*	16%	7%	
* Colorectal*	11%	4%	

Of the patients in the SRS group, 34 (91.9%) had gross total resection and two (8.1%) had subtotal resection. The extent of resection did not differ (p = 0.47) for the WBRT group, with 44 (95.7%) with gross total resection and two (4.3%) with subtotal resection. SRS was performed 26.5 days (mean) after surgery. WBRT was started 20.5 days (mean) after surgery, these results were not different between groups (p = 0.092).

### Overall survival

**Figure 1:**
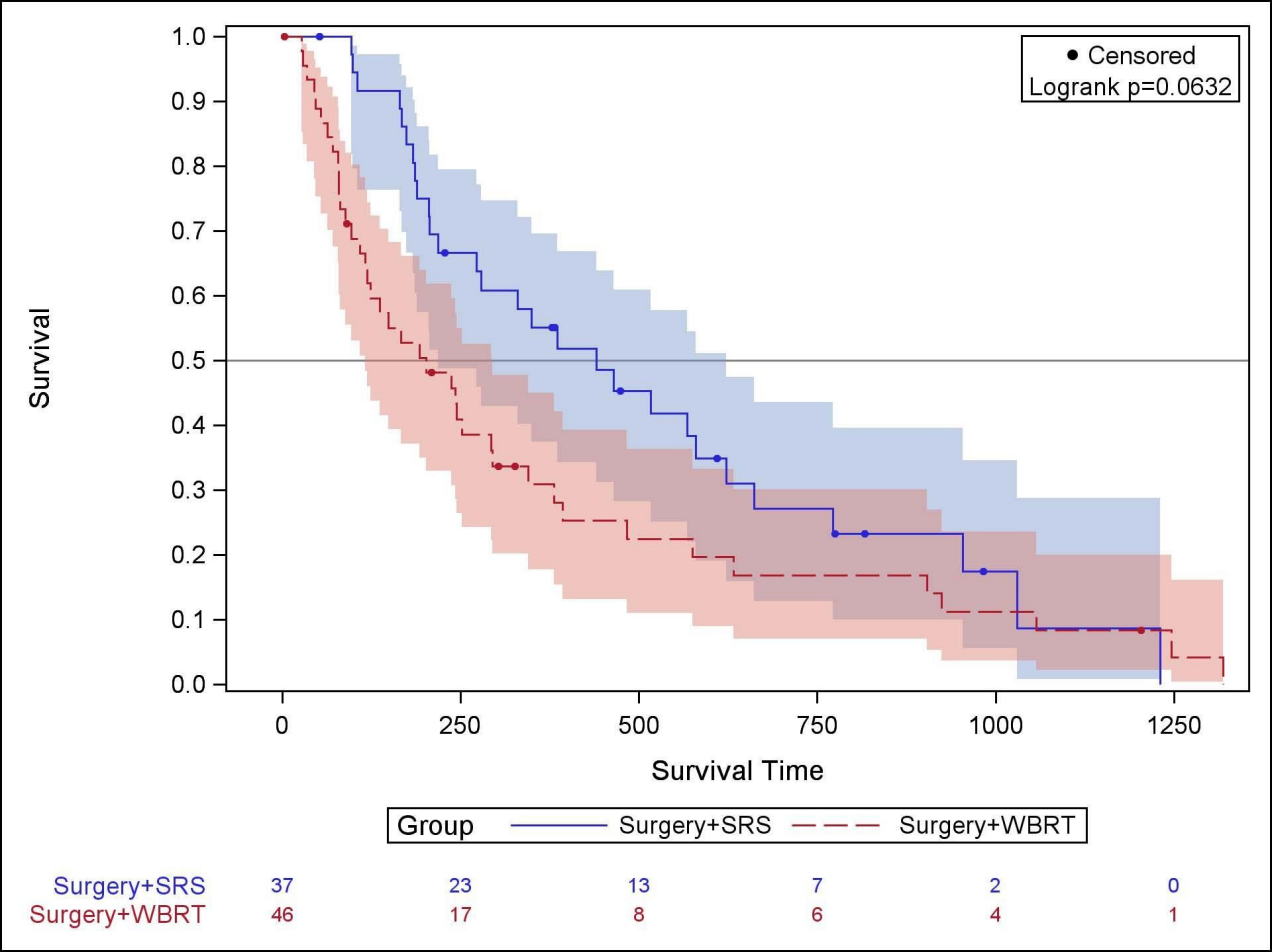
Comparison of survival of SRS and WBRT cohorts Kaplan-Meier survival for surgery followed by SRS to the postoperative (solid line) resection bed compared to surgery followed by WBRT (dashed line). The columns at the bottom of the figure represent the number of surviving patients at each time point.

Median survival was 202 days (CI: 116, 295) for WBRT, and 440 days (CI: 218, 662) for SRS (Figure 1). Both log-rank and Wilcoxon tests were used to compare survival curves. Using the log-rank test, giving equal weight to all time points, overall survival was not statistically different between groups (p = 0.063). However, the Wilcoxon test, weighing early time points more heavily showed improved survival benefit for SRS over WBRT within the first six months (p = 0.0034). Four of 37 (10.8%) SRS patients died within the first six months compared to 15 of 46 (32.6%) WBRT patients.

Multivariate regression was performed using a log-normal model for survival time. Age (p = 0.83), male sex (p = 0.40), and status of systemic disease (p = 0.15) did not differ between groups and were selected as covariates based on their empirical, epidemiological association with poorer prognostic outcomes. The survival benefit conferred by SRS over WBRT remained statistically significant (p = 0.00034).

### Local brain recurrence/control

Same site recurrence at the tumor bed developed in 34% of SRS patients and 55% of WBRT patients over the course of the study, but this difference was not statistically significant (p = 0.093). Local control rate at the tumor bed for SRS was 91.4% at six months, 65.2% at 12 months, and 40% at two years. WBRT local control rates at the tumor bed did not differ significantly, with 75.9%, 64.3% and 16.6% for the same time periods for surviving patients.

### Distant brain recurrence/control

Distant brain recurrence (elsewhere from the surgical bed) occurred in 44% of SRS patients and 40% of WBRT patients (p = 0.74) over the course of the study. Distant control with SRS was similar to local control, with 82.9% at six months, 65.2% at 12 months, and 40% at 24 months. Distant control for WBRT patients was 75.9% at six months, 57.1% at 12 months and 50% at 24 months, not statistically different from SRS distant control. Ten SRS patients (26.1%) had salvage WBRT for local or distant recurrence and seven (15.2%) had salvage SRS. Two WBRT patients (4.3%) had salvage surgery and two (4.3%) had salvage SRS.

### Radiation changes

Radiation-related adverse changes were not statistically more common with SRS (eight of 37; 21.6%) than with WBRT (four of 46; 8.7%) (p = 0.152) (Table 2)). Of the eight SRS patients who developed radiation-related changes, diagnosis was based on CT/PET in seven and surgery in one. Three were treated with observation alone, four were treated with corticosteroids alone, and one was treated with both surgery and corticosteroids. 

**Table 2:** Percentage of patients who developed same site recurrence, distant recurrence within the CNS, and radiation changes stratified by treatment type

	Surgery + SRS n = 37	Surgery + WBRT n = 46	OR(95% CI)	p-Value
Same site recurrence	34%	55%	0.43(0.15-1.16)	0.09
Distant recurrence	44%	40%	1.18(0.44-3.21)	0.74
Radiation changes	28%	10%	3.39(0.81-14.01)	0.081

## Discussion

This study demonstrates that following surgical resection, SRS to the tumor bed has comparable treatment benefit to that of WBRT for patients with brain metastases. Local control, distant central nervous system control, and overall survival were similar. Furthermore, SRS appears to be associated with an early survival advantage. Local radiation-related changes were not significantly increased with SRS.

Our study differs from most other investigations of SRS to the post-resection tumor bed in that we directly compare our postoperative SRS patients to a group of patients treated with postoperative WBRT from the same institution over the same time period. In doing so, we hoped to minimize historical biases. However, we cannot fully account for our own selection bias of patients chosen for SRS or WBRT after their surgical resection. Addressing the issue of selection bias is important, particularly in view of our observation of an early survival advantage for the SRS patients. Unfortunately, the electronic medical record documentation does not adequately provide a rationale for the treatment plan chosen. Factors frequently identified with improved survival, such as age, GPA, and systemic cancer status also did not differ between groups [29-30]. Tumor volume and number of tumors treated were also identified as important prognostic factors [29] which were not significantly different as well. Thus, we cannot determine any patient selection bias for SRS in this retrospective study. Likewise, the reason for the observed early survival advantage is not definable. Even so, the observations that less WBRT patients received salvage therapy and more WBRT patients died within three months of surgery and were excluded from analysis suggests that WBRT patients likely had some degree of more severe disease contributing to selection bias. 

Our results are comparable to those reported in other studies which have examined rates of local control, distant control, survival, and adverse events after SRS to the post-resection tumor bed. The results of these studies are summarized in Table 3 for comparison. The margin dose in our study was similar to the lower end of the spectrum used in the other studies, which make the good results for SRS more compelling. Adverse radiation change in our study appears to be somewhat more frequent with 21.7% of patients having some changes on MRI. Most likely, this result relates to how radiation change was defined. Furthermore, of the eight patients with radiation-related changes in our study, seven (87.5%) were managed with observation or a course of glucocorticosteroids. Only five of 37 (13.9%) required treatment, with only one (2.7%) needing surgical intervention for pathologically confirmed radiation necrosis, a result consistent with reports from other series. Even though the rate of radiation change was not statistically different between SRS and WBRT in this study, a larger series of patients may have demonstrated differences in this regard, so caution is necessary in interpretation.

**Table 3:** Summary of SRS to postoperative tumor bed case series* *              adapted from Table 3 in Sarpong, Litofsky, and Litofsky [7] †          median (in months) ‡           6 months §           12 months ‖           24 months ¶           36 months FU       Follow-up

AUTHOR	# of patients	SRS delivery platform	Margin dose (Gy)	Survival	Local control	Distant control	Adverse radiation effects	Salvage WBRT
Brennan, et al. [12]	40	LINAC frame-based	15 – 22	14.7^†^	78%^§^	56%^§^	17.5%	65.2%
Broemme, et al. [13]	44	LINAC frameless	17 – 18	15.9^†^ 87%^†^; 63.5%^§^	91%^‡^ ; 77%^§^	39%	2.3%	38%
Choi, et al. [14]	120	CyberKnife frameless	20 (median) 10 – 30	17^†^	97% - 84%^§^	65%^‡^; 54%^§^; 38%^‖^	3 – 8%	
Choi, et al. [15]	24	GammaKnife frame based	15 (median)	43.1%^§^; 23.7%^&^	82%^‡^; 71%^§^			
Do, et al. [16]	30	IMRT frame-based	15 – 18	51%^§^	82%^§^	37% total 69%^§^	6.6% (spectroscopy)	47%
Hartford, et al. [17]	47	LINAC frame-based	10 (median) 8 – 20	52.5%^§^ 31.7%^‖^	85%^§^ 66.9%^‖^	44%^§^; 25%^‖^		49%
Iorio-Morin, et al. [6]	110	GammaKnife frame-based	18	11^†^	84%^‡^; 73%^§^; 64%^‖^ 58%^¶^		19% (edema) 32% (enhancement)	28%
Jagannathan, et al. [18]	47	GammaKnife frame-based	19 (mean)	12^†^	94% (14^†^ FU)	38% (5.6^†^ FU)	11% (steroids)	
Jensen, et al. [19]	102	GammaKnife frame-based	17 (median)	10.8^†^ 46.8%^§^	80.8%	35.4%	3% (surgery)	39%
Kalani, et al. [20]	68	GammaKnife frame-based	15	30.2^†^	64.3%			
Karlovits, et al. [21]		LINAC	15 (median)	15.0^†^		56% (16 months)		30.7%
Kelly, et al. [22]	18	LINAC frameless	18 (median) 15 – 18	89%	89% (12.7^†^ FU)	65% (12.7^†^ FU)	0%	
Luther, et al. [23]	120	GammaKnife frame-based	16 (median)	96%^‡^ 87%^§^; 74%^‖^	85.8%	50%	3%	39.2%
Rwigema, et al. [24]	77	Cyberknife frameless	18 (median)	14.5^†^; 91%^‡^; 62.5%^§^; 43.6%^‖^	76.1%^‡^76.1%^§^;74.3%^‖^		2.6%	26%
Soltys, et al. [25]	72	CyberKnife frameless	18 (median) 15 – 30	15.1^†^ 77%^#^; 57%^§^	88%^‡^; 79%^§^	70%^‡^; 47%^§^	9.2% (steroids) 3.9% (surgery)	19%
Steinmann, et al. [26]	33	frameless fractionated	36 – 40	20.2^†^ 64%^§^	30.4^†^ 71%^§^	12.4^†^ 57%^§^	0%	45.4%
Wang, et al. [27]	37	CyberKnife frameless fractionated	24		80%^‡^	80%	5.4%	
PRESENT STUDY	37	LINAC frameless	15 (median)	14.6^†^ 89.2%^‡^	91.4%^‡^; 65.2%^§^; 40%^‖^	82.9%‡; 65.2%^§^; 40%‖	21.7% 10.8% (steroids) 2.7% (surgery)	26.1%

 

The potential value of an SRS treatment plan to the postoperative tumor bed cannot be understated. For patients with metastatic cancer of the brain surviving for longer periods of time after treating their brain disease, long-term quality of life and adverse sequelae associated with treatment are important considerations. Patients frequently express their fears regarding the potential negative effects of whole brain radiation, and studies have shown that long-term survivors of WBRT may incur cognitive and mobility impairments, both of which reduce quality of life. SRS has been shown to be superior to WBRT in terms of reducing cognitive impairment in patients with metastatic cancer to the brain [5, 8-10]. This study provides additional support for equivalence of clinical disease control. Additional work will be necessary to fully ascertain quality of life impacts.

This study has a few limitations. Like all retrospective studies, a number of unaccounted-for confounding factors could be present. The population size is small, which can mask true differences in outcome(s) between treatment groups. The rationale for decision to treat based on physician preference review of patients’ medical records does not readily lend insight into why SRS was selected over WBRT for many of the patients. A selection bias is likely present, and the patients in the SRS and WBRT groups were not matched. Neurocognitive and mobility functions were not formally assessed as outcome measures, thereby missing an opportunity for determination of a superior treatment. Nevertheless, despite these limitations, the study supports clinical equipoise of SRS to the postoperative tumor bed compared to WBRT after surgery.

## Conclusions

Tumor bed SRS can be used to treat brain metastases effectively after surgical resection without significant adverse radiation effects, on par with WBRT. Given the potential for adverse effects of WBRT on cognition and mobility, treatment paradigms should strongly consider SRS to the postoperative tumor bed as the initial step after surgical resection of brain metastases.
